# Exploring innovations, challenges and visions for tomorrow’s healthcare

**DOI:** 10.1016/j.fhj.2024.100214

**Published:** 2024-12-12

**Authors:** Ravina Barrett

**Affiliations:** Associate editor of the Future Healthcare Journal, Royle College of Physicians, London, United Kingdom

As an associate editor of the *Future Healthcare Journal*, I am delighted to present this themed edition dedicated to the future of pharmacy. I am profoundly grateful to Andrew Duncombe for his generous invitation and invaluable support, making this collaboration both a privilege and an honour. This edition comes at a pivotal moment, with further demands and pressures acting on the NHS and the UK health system, which may be replicated globally. Yet, the UK remains a beacon of universal healthcare provision, and maintaining this status is essential.

In this edition, we bring together a diverse group of guest authors, including doctors, patients and pharmacists. They offer intriguing and varied perspectives on healthcare and how pharmacists can play a pivotal role in delivering care that can advance to meet the needs of the UK population. The partnership between physicians and pharmacists is vital in delivering the promise of advanced healthcare, yet it is often fraught with tensions over job roles, descriptions, status and pay, highlighting the need for clear delineation and mutual respect in their collaborative efforts.

We begin with Philip Wiffen *et al*,^1^ both seasoned expert in pain medicine and hospital pharmacy, who has devoted several decades to refining patient care. They provide an overview from a historical perspective of the evolution of the pharmacy’s contribution to healthcare. Their insights into the evolution of pain management and cancer care are both enlightening and essential for understanding the trajectory of pharmacy practice in hospital settings.

Andrew Rochford^2^ contributes a thought-provoking piece on medicine safety and the intricate complexity of care, which intriguingly to me at least presents as chaos theory. His analysis underscores the importance of meticulous attention to detail and the constant vigilance required to ensure patient safety.

Christina Wilson^3^ provides a personal narrative on lifesaving care through the use of injectables and shares her pioneering work in vaccination. Her contributions have been instrumental in advancing care for NHS patients during the pandemic, showcasing the critical role of pharmacists in public health crises such as the COVID-19 pandemic.

From the patient perspective, Marlene Winfield^4^ OBE tackles the challenges of polypharmacy, maintaining drug schedules, and remembering to take medicines on time. She highlights how technology can play a pivotal role in supporting patients, making adherence easier and more reliable.

William Price *et al*^5^ discuss the role of specialisation within hospital settings and how pharmacists can unlock potential for both advanced-care and budget management. Their insights reveal the multifaceted contributions of hospital-based pharmacists in enhancing healthcare delivery and efficiency.

Eugene Catangui^6^ discusses interface care and career progression as a junior pharmacist working in both hospital and academia. The tension, even within the profession, about what further education or accreditation should be undertaken remains ambiguous. He asks, how should one plan their career in such interesting times?

Roshan Tasgaonkar,^7^ offering an international perspective, shares his journey from being a pharmacist in the USA to retraining as a doctor. His story provides valuable insights into the cross-disciplinary benefits and the broader understanding of patient care that such a career transition can offer. Intriguingly, many of the challenges faced by patients and pharmacists in medicines’ supply seem to be universal.

An engaging debate completes this edition, where Steven Williams^8^ articulates the frustrations of many pharmacists and calls for the advancement of professional rights and responsibilities. This is balanced by Imran Rafi’s^8^ counter-argument suggesting that doctors should retain overall responsibility for individual patient care. This debate epitomises the dynamic and evolving dialogue within the healthcare community. Regardless of where one stands on this spectrum of opinion, there is a consensus that modernisation and change are necessary.

With the budget announced by the UK government on 30th of October 2024, it is clear that new fiscal challenges will arise, adding further demands and pressures on the NHS. However, this themed edition of the *Future Healthcare Journal* serves as a testament to the resilience, innovation and forward-thinking that characterise the pharmacy profession and document how the profession can be solution focused. It is through such collective efforts that we can continue to advance and adapt, ensuring that healthcare provision in the UK remains exemplary.

We hope that this edition inspires and informs, sparking new ideas and fostering a deeper understanding of the critical role that pharmacy will play in the future of healthcare.

## Funding

No funding.

## Declaration of competing interest

The authors declare that they have no known competing financial interests or personal relationships that could have appeared to influence the work reported in this paper.
